# Unacylated Ghrelin Improves Vascular Dysfunction and Attenuates Atherosclerosis during High-Fat Diet Consumption in Rodents

**DOI:** 10.3390/ijms20030499

**Published:** 2019-01-24

**Authors:** Michela Zanetti, Gianluca Gortan Cappellari, Andrea Graziani, Rocco Barazzoni

**Affiliations:** 1 Department of Medical Sciences, University of Trieste, 34127 Trieste, Italy; gigici2@iol.it (G.G.C.); barazzon@units.it (R.B.); 2 Department of Translational Medicine, University of Piemonte Orientale “Amedeo Avogadro”, via Solaroli 17, 28100 Novara, Italy; andrea.graziani@med.unipmn.it; 3 Medical School, Università Vita-Salute San Raffaele, via Olgettina 58, 20132 Milano, Italy

**Keywords:** endothelial dysfunction, obesity, ghrelin, nitric oxide, superoxide

## Abstract

Unacylated ghrelin (UnGhr) exerts several beneficial actions on vascular function. The aim of this study was to assess the effects of UnGhr on high-fat induced endothelial dysfunction and its underlying mechanisms. Thoracic aortas from transgenic mice, which were overexpressing UnGhr and being control fed either a standard control diet (CD) or a high-fat diet (HFD) for 16 weeks, were harvested and used for the assessment of vascular reactivity, endothelial nitric oxide synthase (eNOS) expression and activity, thiobarbituric acid reactive substances (TBARS) and glutathione levels, and aortic lipid accumulation by Oil Red O staining. Relaxations due to acetylcholine and to DEA-NONOate were reduced (*p* < 0.05) in the HFD control aortas compared to vessels from the CD animals. Overexpression of UnGhr prevented HFD-induced vascular dysfunction, while eNOS expression and activity were similar in all vessels. HFD-induced vascular oxidative stress was demonstrated by increased (*p* < 0.05) aortic TBARS and glutathione in wild type (Wt) mice; however, this was not seen in UnGhr mice. Moreover, increased (*p* < 0.05) HFD-induced lipid accumulation in vessels from Wt mice was prevented by UnGhr overexpression. In conclusion, chronic UnGhr overexpression results in improved vascular function and reduced plaque formation through decreased vascular oxidative stress, without affecting the eNOS pathway. This research may provide new insight into the mechanisms underlying the beneficial effects of UnGhr on the vascular dysfunction associated with obesity and the metabolic syndrome.

## 1. Introduction

Several lines of evidence suggest that ghrelin, a peptide isolated from the stomach with orexigenic functions, exerts beneficial effects on vascular function and cardiovascular disease [1,2]. In vitro, ghrelin stimulates nitric oxide (NO) production [3,4] and prevents apoptosis of endothelial cells [5,6]. In vivo, it improves endothelial dysfunction in animal models [7] and in humans [8,9] by nitric oxide synthase (NOS)-dependent and -independent mechanisms, particularly by stimulating the release of contractile mediators from the endothelium [10], by antagonizing the effects of systemic vasoconstrictors such as endothelin 1 [11,12], and by mitigating oxidative stress [8].

Most of the circulating ghrelin exists in the unacylated form (UnGhr), which shares some of the beneficial effects of ghrelin on the endothelium and on the vascular system [13], as suggested by clinical studies which have demonstrated an association between low plasma levels of both unacylated and total ghrelin and early atherosclerosis [14,15]. Although the stimulating effects of unacylated ghrelin (UnGhr) on endothelial-dependent vasodilation in isolated arteries from both humans [16] and experimental animals [17,18,19,20] have not been clearly demonstrated, other important properties on vascular endothelium have been definitively confirmed, mechanistically explaining the protective role of UnGhr on the vascular system during disease states. Although no specific receptor has yet been identified, in vitro UnGhr protects endothelial cells from oxidative stress [21,22] and prevents diabetes-induced endothelial progenitor cell damage by modulating eNOS activity and reactive oxygen species formation [23]. In addition to the direct beneficial effects on endothelial cells, studies in experimental models of obesity have shown that unacylated ghrelin counteracts systemic insulin resistance, oxidative stress, and inflammation, which are typically associated with vascular disease [24]. For these reasons, the administration of unacylated ghrelin has been proposed as a therapeutic tool for the treatment of obesity-related metabolic and cardiovascular complications [25,26]. Consistent with these findings, obese subjects with the metabolic syndrome, who are also typically predisposed to cardiovascular complications, are characterized by a low unacylated to acylated ghrelin ratio [27]. Although the available evidence convincingly suggests that potential benefits of UnGhr on high-fat diet (HFD)-induced endothelial dysfunction and early vascular disease exist, no information is available on the impact of UnGhr on endothelial vasorelaxation, eNOS function, vascular oxidative stress and atherosclerosis burden in the setting of obesity and insulin resistance.

We therefore studied a transgenic mouse model of systemic UnGhr overproduction to test the hypotheses that UnGhr: (1) positively impacts HFD-induced impaired vasorelaxation by enhancing eNOS expression/function and/or reducing vascular oxidative stress; (2) lowers the burden of initial atherosclerotic vascular remodeling in the aortic wall.

## 2. Results

### 2.1. Body Weight and Glucose Levels

Body weight was comparable between the groups fed the control diet and the high-fat diet (Table 1), as previously reported [24]. Blood glucose was also similar during the control diet; in contrast, after the high-fat diet it increased (*p* < 0.05) in the wild type but not in the UnGhr group of animals, as previously published [24] (Table 1).

### 2.2. Vascular Reactivity

**Figure 1:**
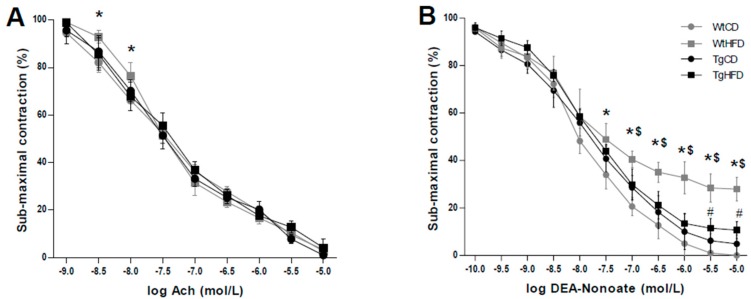
(**A**) Concentration-response curves to acetylcholine in wild type (Wt) and transgenic mice (Tg) overexpressing unacylated ghrelin fed a control (CD) or a high-fat diet (HFD). Vascular reactivity studies were performed on aortic segments from each group. * *p* ≤ 0.05 WtHFD vs. WtCD. *n* = 7 animals/group. Contractions to phenylephrine were similar among the experimental groups. (**B**) Concentration-response curves to DEA-NONOate in the same groups. * *p* ≤ 0.05 vs. WtCD. ^$^ *p* < 0.05 TgHFD vs. WtHFD. # *p* < 0.05 TgHFD vs. WtCD. Contractions to phenylephrine were not significantly different in the four groups. *n* = 7 animals/group.

During the control diet (CD), endothelium-dependent vasodilation was similar within the aortas from lean wild type and UnGhr-overexpressing animals. As expected, compared with those from lean animals on the CD, vessels from obese, HFD-fed control mice displayed impaired (*p* < 0.05) endothelial vasorelaxation in response to acetylcholine (Figure 1A). In contrast, endothelium-dependent vasodilation in response to acetylcholine was not impaired in the aortas from obese, HFD-fed UnGhr-overexpressing animals (Figure 1A). Endothelium-independent vasorelaxation in response to DEA-NONOate was similar among experimental genotypes during the control diet (Figure 1B). During the HFD, impaired (*p* < 0.05) vasodilation in response to DEA-NONOate in the aortas from obese wild type mice was normalized by the overexpression of unacylated ghrelin in the transgenic group under the same experimental conditions (Figure 1B). Contractions in response to phenylephrine did not differ among groups.

### 2.3. Aortic eNOS Expression and Activity

**Figure 2:**
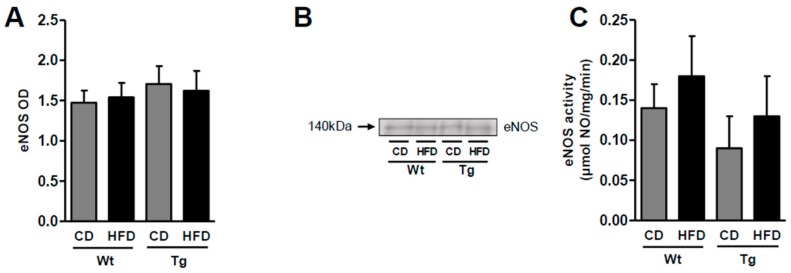
Protein expression, as detected by Western blot analysis, of the total endothelial nitric oxide synthase (eNOS) (**A**) with representative blots (**B**) and nitric oxide synthase (NOS) activity (**C**) in aortas from control (Wt) and transgenic (Tg) mice fed the control diet (CD) or a high-fat diet (HFD). The data represent seven mice per group. OD: optical density.

The eNOS expression was comparable in aortas from wild type and UnGhr-overexpressing transgenic animals, both under the control and high-fat diet conditions (Figure 2A,B). Accordingly, eNOS activity was not different between vessels from lean and obese HFD-fed animals from both genotypes (Figure 2B).

### 2.4. Systemic and Vascular Oxidative Stress and Antioxidant Potential

**Figure 3:**
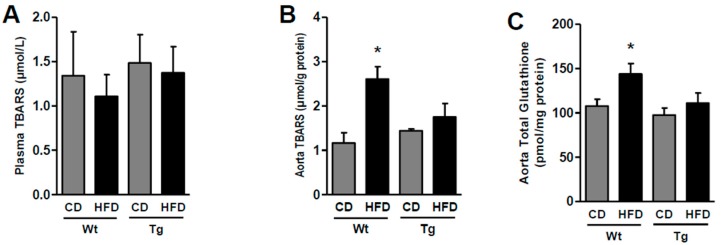
Systemic and vascular oxidative stress and antioxidant potential. Quantitative analysis of plasma (**A**) and aortic (**B**) thiobarbituric acid reactive substances (TBARS) and tissue total glutathione (**C**) in control (Wt) and transgenic (Tg) mice fed the control diet (CD) or the high-fat diet (HFD). The data represent seven mice per group. * *p* < 0.05 vs. other groups.

Since oxidative stress is associated with endothelial dysfunction, levels of thiobarbituric acid reactive substances (TBARS) were assessed in plasma and in aortas. Similar concentrations of plasma TBARS were found in the wild type and UnGhr-overexpressing mice, both under the control and high-fat diets (Figure 3A). Aortic levels of TBARS were not different in vessels from animals belonging to the two experimental genotypes under the control diet; during the HFD, TBARS levels increased (*p* < 0.05) in aortas from wild type mice, but not in vessels from obese UnGhr animals (Figure 3B). A similar trend was observed for aortic glutathione (Figure 3C).

### 2.5. Early Atherosclerosis Burden in Mouse Aorta

**Figure 4:**
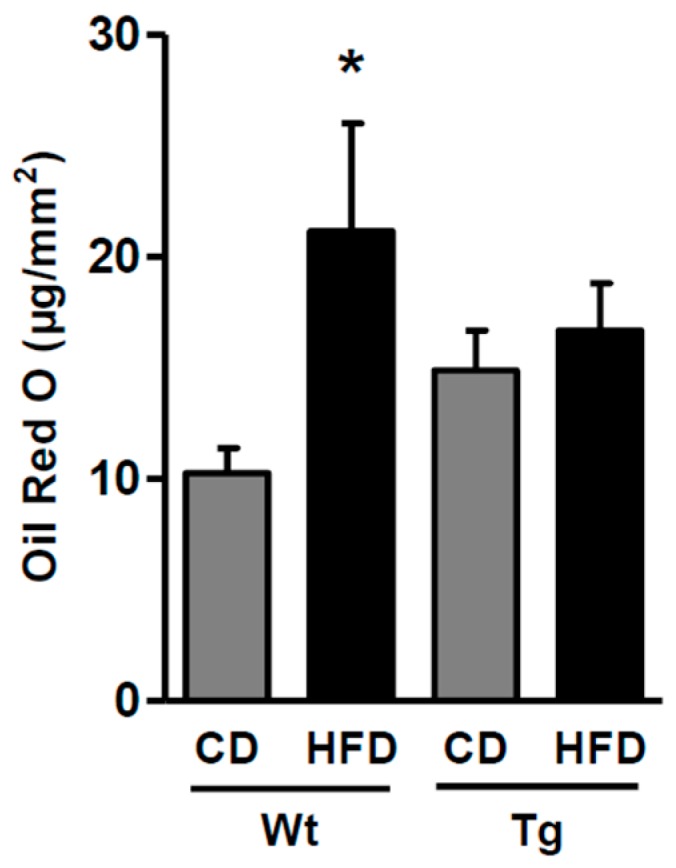
Lipid accumulation quantified by Oil Red O (ORO) staining in the aortas from control (Wt) and transgenic (Tg) mice fed the control diet (CD) or the high-fat diet (HFD). * *p* < 0.05 vs. WtCD.

Early atherosclerotic plaque burden was measured in thoracic aortas by quantifying lipid deposition using Oil Red O staining. During the CD, lean animals showed similar lipid deposition; the high-fat diet resulted in increased (*p* < 0.05) lipid deposition in aortas from wild type mice, which was prevented by UnGhr overexpression under the same dietary regimen (Figure 4).

## 3. Discussion

In this study, we demonstrate that systemic UnGhr upregulation, resulting in sustained levels of the circulating peptide, prevents high-fat diet-induced vascular dysfunction to endothelium-dependent and -independent stimuli. This effect is not likely mediated by enhanced eNOS expression and function, but by reduced vascular oxidative stress, resulting in the prevention of early atherosclerosis and lipid accumulation in the vascular wall. To the best of the authors’ knowledge, the present study is the first to examine the effects of unacylated ghrelin on vascular tone and oxidative stress in a transgenic model of systemic circulating unacylated ghrelin upregulation and high-fat diet-induced obesity.

Previous reports have addressed the issue of the vascular response to unacylated ghrelin in different species, vascular districts, and disease states. Collectively, the majority of these studies have shown that unacylated ghrelin evokes endothelium-dependent dilation. Under physiological conditions, this has been shown in systemic arteries from humans [16], in coronary arteries from pigs [18], in systemic vessels from rats [17], and in the cerebral circulation of mice [19]. In this latter model, however, Ku et al. failed to demonstrate any vasodilator effect of unacylated ghrelin on the isolated thoracic aorta, as well as on nitric oxide and superoxide production [20]. The apparent discrepancy between these data and those from our study is likely related to the different metabolic milieu in the two models. As previously demonstrated [24], the obese UnGhr-overexpressing mice used in this study were characterized by a better metabolic profile and by lower insulin resistance than the respective group on the high-fat diet; we suggest that this may have influenced vascular responses to acetylcholine in the thoracic aortas. In accordance with these findings, it has been previously demonstrated that obesity and insulin resistance are relevant determinants of vascular function in humans, playing a far more important role than isolated dysglycemia [28].

In addition, we found that sustained UnGhr overexpression was associated with lower oxidative stress in the aorta. Indeed, many studies in animal models have shown an effect of unacylated ghrelin on the redox state [29,30], indicating that in normal conditions the peptide protects vessels from oxidative stress, both by inducing the expression of endogenous antioxidants [22] and by quenching the activity of NADPH oxidase [19]. Similar results have been obtained in patients in the setting of hypertension and diabetes [8,23]. Consistent with the modulatory effect of unacylated ghrelin on oxidative stress, we found that vessels from transgenic mice were resistant to high-fat diet-induced oxidative damage, as demonstrated by the lack of an increase of TBARS and of glutathione in this group. Notably, although blood glucose was lower in HFD-induced obese UnGhr-overexpressing mice, systemic oxidative stress was unaffected, suggesting that the reduced vascular damage observed in these conditions is selectively associated with unacylated ghrelin overexpression.

Consistent with the study by Ku et al. [20], who failed to demonstrate UnGhr-stimulated nitric oxide production of mouse systemic arteries, no evidence was found of any effect of UnGhr overexpression on eNOS protein levels and activity in mouse thoracic aortas during both the control and the high-fat diet. The effects of obesity on eNOS expression and activity have been previously assessed in different experimental models with mixed results [31,32]. In vivo, evidence in humans shows that obesity-induced endothelial dysfunction is mainly associated with impaired NO production and eNOS expression [33]. However, severely obese individuals are paradoxically protected by endothelial dysfunction compared with obese subjects [34]. Therefore, different species, experimental models, and lengths of exposure to test diets could explain these discrepant results. Further studies will be necessary to unravel the underlying mechanisms.

Previous in vivo studies in humans have suggested that the combination of both impaired endothelial-dependent and -independent vasodilation is a stronger predictor of cardiovascular events than endothelial dysfunction in response to acetylcholine alone [35,36] and that vasodilation in response to NO donors is a useful marker for the assessment of the degree of atherosclerosis [37]. This observation holds particularly true in the presence of a low degree of atherosclerosis [38]. Consistent with these findings, impaired vasorelaxation in response to both acetylcholine and to the NO donor DEA-NONOate and higher lipid deposition were detected in aortas from obese mice belonging to the wild type genotype. In contrast, UnGhr overexpression prevented HFD-induced endothelium-dependent and -independent dysfunction and lipid accumulation in aortas from transgenic mice. The evidence from animal studies for the direct involvement of unacylated and total ghrelin on early plaque development is not equivocal [39,40], while beneficial effects on plaque stability, via different mechanisms, have been demonstrated for both peptides [41,42]. Collectively, our findings suggest that the systemic upregulation of unacylated ghrelin is associated with reduced vascular wall remodeling and early atherosclerosis development in the setting of obesity and insulin resistance. Although we cannot rule out a direct effect of unacylated ghrelin on plaque development and progression, we suggest that the beneficial metabolic actions of the peptide could have contributed to its favorable effects on the vascular system.

Oil Red O (ORO) is commonly used to quantify atherosclerosis in mice [43,44] and the specificity of this dye for lipids is also demonstrated by its use in advanced modified techniques in several settings [45,46]. In this study, ORO staining and the following lipid measurements were performed in rings from thoracic aortas and not on histological sections. This method was developed and used by other authors as well [47,48]. In addition, both Andres-Manzano et al. [43] and Lin et al. [44] showed an excellent agreement between aorta ORO staining and other conventional histopathology techniques used for atherosclerosis lesion assessment. Although the technique does not allow the precise lipid composition of the lesions to be defined, it does permit a comparative assessment of the effects of HFD and UnGhr on plaque development.

In conclusion, unacylated ghrelin demonstrates positive effects on vascular function and early atherosclerosis development associated with high-fat diet-induced obesity. Underlying mechanisms include directly reduced vascular oxidative stress and lipid deposition within the aorta and, indirectly, a favorable metabolic pattern characterized by reduced systemic insulin resistance. Although the relative contribution of each of these actions to the preservation of vascular function cannot be directly extrapolated from the current findings, the results of this study provide a rationale for the therapeutic use of unacylated ghrelin in the treatment of vascular dysfunction and cardiovascular disease in conditions associated with altered peptide levels, such as obesity and insulin resistance.

## 4. Material and Methods

### 4.1. Animal Protocol

The generation and characteristics of transgenic mice overexpressing UnAG (Tg Myh6/Ghrl) have been described previously [49]. Selective overproduction of ghrelin in the heart, characterized by negligible acylating activity, results in a 40-fold increment in circulating UnAG without acylated ghrelin (AG) modification. Experiments were approved by the Institutional Review Board at the Department of Medicine, University of Piemonte Orientale (Italy). All experiments were conducted on 14 young adult male Tg Myh6/Ghrl and 14 wild type male mice, matched for age and weight. Animals underwent 16-week standard or HFD feeding (10% or 60% calories from fat; Research Diets, New Brunswick, NJ, USA) and were killed as described previously [24]. This study was part of a larger protocol aimed at assessing the effects of unacylated ghrelin on HFD-induced metabolic alterations, inflammation, and oxidative stress [24].

### 4.2. Analytical Methods

#### 4.2.1. Plasma Glucose

Plasma glucose levels were determined by standard colorimetric assay [50].

#### 4.2.2. Plasma and Aortic Oxidative Stress and Antioxidant Potential

Oxidative stress in plasma and aortas was determined by measuring lipid peroxidation (TBARS), using a commercially available kit (Oxitek, ZeptoMetrix Co., Buffalo, NY, USA) as previously described [51]. In addition, total glutathione was determined in aortas as referenced [52].

#### 4.2.3. Analysis of Vascular Reactivity

Endothelium-dependent and -independent vasodilation were assessed in organ chambers [53]. Briefly, rings (4 mm long) from thoracic ascending aortas were used for assessing vascular reactivity. The rings were suspended in organ chambers filled with 25 mL of gassed (95% O_2_ and 5% CO_2_) modified Krebs-Ringer bicarbonate solution (pH 7.4, temperature 37 °C; composition in mmol/L: 118.3 NaCl, 4.7 KCl, 2.5 CaCl_2_, 1.2 MgSO_4_, 1.2 KH_2_PO_4_, 25.0 NaHCO_3_, 0.026 calcium sodium EDTA, and 11.1 glucose). The rings were allowed to equilibrate for 1 h and then were stretched to the optimal point on the length–tension curve, as determined by repeated exposure to 20 mmol/L KCl. The maximal contraction of each ring was determined by phenylephrine 10^−5^ mol·L^−1^. Submaximal contractions were obtained using a 10^−6^ to 10^−7^ mol·L^−1^ concentration of phenylephrine. Acetylcholine (10^−9^ to 10^−5^ mol/L) was added cumulatively during a submaximal contraction to phenylephrine. Concentration responses to DEA-NONOate (10^−10^ to 10^−5^ mol·L^−1^) were similarly performed.

### 4.3. Assessment of NOS Expression and Activity in the Aorta

Protein levels of eNOS were analyzed by Western blot analysis as previously described [50,51,53]. Aortic segments were isolated and stored in liquid nitrogen. The segments were pulverized and resuspended in lysis buffer (50 mmol/L Tris HCl, 0.1 mmol/L EDTA, 0.1 mmol/L EGTA, 0.1% SDS, 0.1% deoxycholate, 1% Igepal, 2 µg/mL leupeptin, 2 µg/mL aprotinin, 1 mmol/L PMSF, 1 µg/mL pepstatin). Aortic debris was homogenized on ice, centrifuged at 14,000 rpm for 10 min, and the protein concentration was assessed by the bicinchoninic acid protein assay reagent (BCA Protein Assay, Pierce, Rockford, IL, USA). Protein weighing 20 µg was loaded on 4% stacking/9% separating SDS/PAGE. The resolved proteins were transferred to a 0.2-µm nitrocellulose membrane on a semi-dry electrophoretic transfer system (Bio-Rad) for Western blot analysis. Equal protein load was confirmed by Ponceau staining. Blots were blocked and incubated with an anti-eNOS antibody (1:1000, BD Transduction Laboratories Franklin Lakes, NJ, USA) overnight at 4 °C. After extensive washing, horseradish peroxidase-linked secondary antibody was added (1:1000, Jackson Laboratories, West Grove, PA, USA). Chemiluminescence detection was performed with the Western blotting ECL system (Amersham, Little Chalfont, Buckinghamshire, UK) and exposed to X-ray films. The autoradiographs were analyzed by densitometry (Model GS-700, Biorad, Hercules, CA, USA). Total NOS activity in aortas was measured using the oxyhemoglobin to methemoglobin conversion assay as previously described [51].

### 4.4. Measurement of Atherosclerotic Burden in Aortas

Quantification of lipid deposition on the surface of aortic arches (fixed for 24 h in 10% formalin) was determined by Oil Red O (ORO) staining as previously described [48]. The thoracic aortas, cleared from any perivascular adipose tissue, were incubated with Oil Red O solution (Oil Red O 3% (*w*/*v*) was dissolved in isopropanol at 56 °C for 60 min, the solution was then filtered and diluted 2:1 with ultrapure water) for 30 min at room temperature on a shaker. Samples were then washed by soaking for 10 s in 70% isopropanol solution, followed by soaking with water. After a 10-min incubation in chloroform/methanol (2:1 *v*/*v*) and the removal of the aortic segments, the absorbance of the solution, proportional to the amount of lipid in each sample, was determined spectrophotometrically at 520 nm. The endothelial surface in the sample was measured by a high-resolution scan of the opened and flattened aortic segments and the relative area was calculated by dedicated software (ImageJ, NIH). The results were expressed as µg ORO/mm^2^.

### 4.5. Statistical Analysis

Statistical analysis was performed with SPSS (version 17, IBM, Armonk, NY, USA). The groups were compared using a *t*-test or ANOVA followed by post-hoc tests. Benjamini–Hochberg correction for multiple comparisons was applied. Quantitative data are presented as means ± S.E.M. A *p*-value <0.05 was considered statistically significant.

## Figures and Tables

**Table 1:** Characteristics of study animals.

Parameter	Tg UnGhr	Wt
Weight (g)		
16-week CD	28.7 ± 2.1	31 ± 2.1
16-week HFD	36.6 ± 1.1 *	37.9 ± 3 *
Blood glucose (mg/dL)		
16-week CD	98.2 ± 8	106.0 ± 7.3
16-week HFD	102 ± 11.6	161.9 ± 30.7 *^,#^

Tg UnGhr: transgenic mice overexpressing unacylated ghrelin; Wt: wild type mice; CD: control diet; HFD: high-fat diet. * *p* < 0.05 vs. the same parameter and group on the CD; ^#^ *p* < 0.05 vs. the other group on the HFD. *n* = 7 animals/group.
